# Supplementary material for: Characterization of entomological drivers of malaria transmission in five villages, Keerom Regency, Papua, Indonesia
Source: PLoS One. 2026 Jun 30;21(6):e0339951. doi: 10.1371/journal.pone.0339951 (PMC13318021; doi:10.1371/journal.pone.0339951)
Supplement: S1 File — (DOCX) [file pone.0339951.s001.docx]

# Supplementary Files 1

Table S1-1. Household survey results

| **Villages** | **Sanggaria** | **Yaturaharja** | **Ubiyau** | **Sawanawa** | **Pitewi** | **Total** |
| --- | --- | --- | --- | --- | --- | --- |
|  | **n=76** | **n=49** | **n=29** | **n=17** | **n=30** | **n=201** |
|  |  |  |  |  |  |  |
|  |  |  |  |  |  |  |
| 1. **Interviewee** | | | | | | |
|  |  |  |  |  |  |  |
| **Gender** |  |  |  |  |  |  |
| Male | 46 (60.5%) | 15 (30.6%) | 8 (27.6%) | 8 (47.1%) | 11 (36.7%) | 88 (43.8%) |
| Female | 30 (39.5%) | 34 (69.4%) | 21 (72.4%) | 9 (52.9%) | 19 (63.3%) | 113 (56.2%) |
| **Age (years)** | 45.9 (± 13.5) | 41.7 (± 12.0) | 40.0 (± 13.8) | 35.9 (± 10.3) | 36.5 (± 13.7) | 41.8 (± 13.4) |
| Range | 18 - 78 | 15 - 72 | 17 - 65 | 17 - 55 | 18 - 77 | 15 - 78 |
| **Relationship** |  |  |  |  |  |  |
| Head of household (or his/her spouse) | 94.74% | 91.84% | 96.55% | 100.00% | 83.33% | 93.03% |
| Other core family | 3.95% | 4.08% | 3.45% | 0.00% | 16.67% | 5.47% |
| Not core family | 1.32% | 4.08% | 0.00% | 0.00% | 0.00% | 1.49% |
| **Last Education** |  |  |  |  |  |  |
| No education | 0.0% | 0.0% | 10.3% | 23.5% | 3.3% | 4.0% |
| Not finish primary school | 6.6% | 22.4% | 0.0% | 0.0% | 10.0% | 9.5% |
| Primary and Junior high | 53.9% | 42.9% | 37.9% | 64.7% | 6.7% | 42.8% |
| Senior high or above | 39.5% | 34.7% | 51.7% | 11.8% | 80.0% | 43.8% |
| **Livelihood** | **n=79** | **n=50** | **n=29** | **n=17** | **n=37** | **n=212** |
| Farmer | 39.2% | 30.0% | 34.5% | 70.6% | 29.7% | 37.3% |
| Breeder | 3.8% | 0.0% | 3.4% | 0.0% | 8.1% | 3.3% |
| Merchant | 12.7% | 28.0% | 0.0% | 0.0% | 27.0% | 16.0% |
| Woodcutter | 0.0% | 0.0% | 6.9% | 11.8% | 5.4% | 2.8% |
| Laborer-technician | 27.8% | 6.0% | 17.2% | 0.0% | 2.7% | 14.6% |
| Professional | 2.5% | 2.0% | 0.0% | 0.0% | 0.0% | 1.4% |
| Civil servant | 11.4% | 10.0% | 0.0% | 0.0% | 18.9% | 9.9% |
| Other | 2.5% | 10.0% | 13.8% | 17.6% | 8.1% | 8.0% |
| No job | 0.0% | 14.0% | 24.1% | 0.0% | 0.0% | 6.6% |
| 1. **Households** | | | | | | |
|  |  |  |  |  |  |  |
| **Household members** |  |  |  |  |  |  |
| Total members | 3.8 (± 1.6) | 3.8 (± 1.3) | 4.8 (± 2.4) | 4.8 (± 2.1) | 4.1 (± 2.0) | 4.1 (± 1.8) |
| members ≤ 5 years | 0.3 (± 0.6) | 0.5 (± 0.7) | 1.0 (± 1.2) | 0.6 (± 0.7) | 0.4 (± 0.6) | 0.5 (± 0.8) |
| Total de-facto members | 3.5 (± 1.6) | 3.7 (± 1.3) | 4.7 (± 2.4) | 4.8 (± 2.1) | 3.4 (± 1.5) | 3.8 (± 1.8) |
| **Wealth index** |  |  |  |  |  |  |
| 1st Quantile | 7.9% | 0.0% | 24.1% | 35.3% | 13.3% | 11.4% |
| 2nd Quantile | 17.1% | 18.4% | 44.8% | 11.8% | 23.3% | 21.9% |
| 3rd Quantile | 21.1% | 26.5% | 20.7% | 35.3% | 20.0% | 23.4% |
| 4th Quantile | 28.9% | 40.8% | 10.3% | 11.8% | 33.3% | 28.4% |
| 5th Quantile | 25.0% | 14.3% | 0.0% | 5.9% | 10.0% | 14.9% |
| **Wall material** |  |  |  |  |  |  |
| Wooden board + half cement half wooden board | 30.26% | 30.61% | 13.79% | 11.76% | 50.00% | 29.35% |
| Cement + concrete + stone | 69.74% | 67.35% | 86.21% | 88.24% | 50.00% | 70.15% |
| Others | 0.00% | 2.04% | 0.00% | 0.00% | 0.00% | 0.50% |
| **Roof material** |  |  |  |  |  |  |
| Metal (iron sheeting) | 97.37% | 95.92% | 100.00% | 100.00% | 96.67% | 97.51% |
| Others | 2.63% | 4.08% | 0.00% | 0.00% | 3.33% | 2.49% |
| **Floor material** |  |  |  |  |  |  |
| Wooden board | 13.16% | 18.37% | 13.79% | 11.76% | 46.67% | 19.40% |
| Ceramic | 50.00% | 34.69% | 37.93% | 29.41% | 46.67% | 42.29% |
| Cement | 36.84% | 46.94% | 48.28% | 58.82% | 3.33% | 37.81% |
| Others | 0.00% | 0.00% | 0.00% | 0.00% | 3.33% | 0.50% |
| **Floor height** |  |  |  |  |  |  |
| **· Wooden board floor** |  |  |  |  |  |  |
| Groundlevel | 70.00% | 11.11% | 25.00% | 100.00% | 0.00% | 28.21% |
| 0 > and ≤ 100 cm | 20.00% | 77.78% | 25.00% | 0.00% | 50.00% | 43.59% |
| More than 100 cm | 10.00% | 11.11% | 50.00% | 0.00% | 50.00% | 28.21% |
| **· Others** |  |  |  |  |  |  |
| Groundlevel | 87.88% | 25.00% | 100.00% | 93.33% | 12.50% | 67.28% |
| 0 > and ≤ 100 cm | 10.61% | 75.00% | 0.00% | 6.67% | 87.50% | 32.10% |
| More than 100 cm | 1.52% | 0.00% | 0.00% | 0.00% | 0.00% | 0.62% |
|  |  |  |  |  |  |  |
|  |  |  |  |  |  |  |
| **Door** |  |  |  |  |  |  |
| Average | 2.5 (± 0.8) | 2.4 (± 0.5) | 2.1 (± 0.4) | 2.0 (± 0.0) | 2.3 (± 0.5) | 2.3 (± 0.6) |
| House with screened doors | 1.3% | 0.0% | 0.0% | 0.0% | 46.7% | 7.5% |
| **Window** |  |  |  |  |  |  |
| Average | 7.2 (± 4.2) | 3.7 (± 3.1) | 4.7 (± 1.5) | 5.5 (± 1.5) | 6.1 (± 3.3) | 5.7 (± 3.6) |
| House with screened windows | 5.3% | 0.0% | 0.0% | 0.0% | 50.0% | 9.5% |
| **Eave** |  |  |  |  |  |  |
| Eave exist | 98.7% | 89.8% | 100.0% | 100.0% | 76.7% | 93.5% |
| Partly Screened | 30.7% | 4.5% | 0.0% | 35.3% | 4.3% | 17.0% |
| Screened | 21.3% | 4.5% | 0.0% | 0.0% | 69.6% | 18.1% |
| **Electricity** |  |  |  |  |  |  |
| No electricity | 0.0% | 0.0% | 3.4% | 0.0% | 0.0% | 0.5% |
| Have electricity | 100.0% | 100.0% | 96.6% | 100.0% | 100.0% | 99.5% |
| State Electricity Company (PLN) | 100.0% | 100.0% | 100.0% | 100.0% | 100.0% | 100.0% |
| Solar Cell | 0.0% | 0.0% | 0.0% | 0.0% | 0.0% | 0.0% |
| Generator | 0.0% | 0.0% | 0.0% | 0.0% | 0.0% | 0.0% |
| 1. **Suffering of malaria** | | | | | | |
|  |  |  |  |  |  |  |
| **At least one member of the household had suffered by malaria** | 25.0% | 75.5% | 93.1% | 64.7% | 50.0% | 54.2% |
| Nuclear family members | 96.9% | 98.2% | 100.0% | 100.0% | 88.9% | 96.9% |
| Not nuclear family members | 3.1% | 1.8% | 0.0% | 0.0% | 11.1% | 3.1% |
| Answer not filled | 0.0% | 0.0% | 0.0% | 0.0% | 0.0% | 0.0% |
| **Malaria last case** |  |  |  |  |  |  |
| ≤ 2 weeks ago | 10.5% | 8.1% | 11.1% | 0.0% | 6.7% | 8.3% |
| > 2 weeks ago and ≤ one month | 21.1% | 8.1% | 37.0% | 63.6% | 40.0% | 27.5% |
| > one month | 63.2% | 81.1% | 51.9% | 36.4% | 40.0% | 60.6% |
| Don't know | 5.3% | 2.7% | 0.0% | 0.0% | 13.3% | 3.7% |
| **How to know malaria** |  |  |  |  |  |  |
| Physician's examination result | 12.5% | 2.0% | 0.0% | 0.0% | 19.0% | 5.2% |
| Microscopy or RDT result | 75.0% | 68.6% | 63.4% | 56.3% | 66.7% | 66.7% |
| Symptoms | 4.2% | 23.5% | 36.6% | 43.8% | 9.5% | 24.2% |
| Previous experience | 8.3% | 5.9% | 0.0% | 0.0% | 0.0% | 3.3% |
| Notified by someone else | 0.0% | 0.0% | 0.0% | 0.0% | 0.0% | 0.0% |
| Other | 0.0% | 0.0% | 0.0% | 0.0% | 0.0% | 0.0% |
| Don't know | 0.0% | 0.0% | 0.0% | 0.0% | 4.8% | 0.7% |
| **Death caused by malaria in recent 2 years** | 5.3% | 0.0% | 0.0% | 0.0% | 0.0% | 2.0% |
| 1. **Mosquito biting prevention** | | | | | | |
|  |  |  |  |  |  |  |
| **IRS** |  |  |  |  |  |  |
| Have been sprayed | 38.2% | 30.6% | 86.2% | 0.0% | 16.7% | 36.8% |
| Sprayed < 3 months | 0.0% | 0.0% | 68.0% | - | 0.0% | 23.0% |
| Sprayed 3-6 months | 0.0% | 13.3% | 32.0% | - | 20.0% | 14.9% |
| Sprayed > 6 months | 100.0% | 86.7% | 0.0% | - | 80.0% | 62.1% |
| Sprayed by health workers | 58.6% | 93.3% | 8.0% | - | 100.0% | 51.4% |
| Sprayed by NGO | 31.0% | 0.0% | 92.0% | - | 0.0% | 43.2% |
| Sprayed by others | 6.9% | 0.0% | 0.0% | - | 0.0% | 2.7% |
| **ITN** |  |  |  |  |  |  |
| Bedroom average | 2.4 (± 0.9) | 2.5 (± 0.9) | 2.5 (± 1.2) | 2.4 (± 1.2) | 2.2 (± 0.8) | 2.4 (± 0.9) |
| Average bednet coverage | 2.3 (± 1.1) | 2.3 (± 0.8) | 2.8 (± 1.4) | 2.8 (± 1.5) | 2.4 (± 1.2) | 2.4 (± 1.1) |
| Everybody using net | 88.2% | 65.3% | 89.7% | 76.5% | 53.3% | 76.6% |
| Slept under net lastnight | 3.1 (86.4%) | 2.9 (80.3%) | 4.6 (94.2%) | 4.5 (92.0%) | 2.9 (78.9%) | 3.4 (85.4%) |
| ITN Access | 85.1% | 79.9% | 92.6% | 95.1% | 83.5% | 86.1% |
| **Households with ITNs questions** | **n = 72** | **n = 45** | **n = 28** | **n = 17** | **n = 26** | **n = 188** |
| **Origin of ITNs** |  |  |  |  |  |  |
| Healthcare (antenatal care) | 5 (6.9%) | 2 (4.4%) | 6 (21.4%) | 0 (0.0%) | 0 (0.0%) | 13 (6.9%) |
| Mass distribution | 66 (91.7%) | 43 (95.6%) | 0 (0.0%) | 0 (0.0%) | 25 (96.2%) | 134 (71.3%) |
| Non-government organization | 1 (1.4%) | 0 (0.0%) | 22 (78.6%) | 17 (100.0%) | 0 (0.0%) | 40 (21.3%) |
| **ITNs age** |  |  |  |  |  |  |
| > 3 years | 2 (2.8%) | 0 (0.0%) | 0 (0.0%) | 0 (0.0%) | 0 (0.0%) | 2 (1.1%) |
| > 1 year and ≤ 3 years | 19 (26.4%) | 10 (22.2%) | 0 (0.0%) | 0 (0.0%) | 0 (0.0%) | 29 (15.4%) |
| ≤ 1 year | 51 (70.8%) | 35 (77.8%) | 28 (100.0%) | 17 (100.0%) | 26 (100.0%) | 157 (83.5%) |
| **ITNs condition** |  |  |  |  |  |  |
| Lots of holes | 0 (0.0%) | 0 (0.0%) | 0 (0.0%) | 0 (0.0%) | 0 (0.0%) | 0 (0.0%) |
| Few holes | 6 (8.3%) | 4 (8.9%) | 0 (0.0%) | 0 (0.0%) | 0 (0.0%) | 10 (5.3%) |
| Good | 66 (91.7%) | 39 (86.7%) | 28 (100.0%) | 17 (100.0%) | 26 (100.0%) | 176 (93.6%) |
| **Insect repellent** |  |  |  |  |  |  |
| Using repellents indoor | 72.4% | 59.2% | 62.1% | 23.5% | 96.7% | 67.2% |
| Mosquito coil | 43.6% | 69.0% | 94.4% | 100.0% | 65.5% | 62.2% |
| Mosquito spray | 58.2% | 34.5% | 5.6% | 25.0% | 62.1% | 45.9% |
| Emanator | 20.0% | 0.0% | 0.0% | 0.0% | 48.3% | 18.5% |
| Personal repellent | 20.0% | 6.9% | 0.0% | 0.0% | 13.8% | 12.6% |
| 1. **Human behavior at night** | | | | | | |
|  |  |  |  |  |  |  |
| **Dinner time** |  |  |  |  |  |  |
| Before 19.00 | 31.6% | 34.7% | 31.0% | 76.5% | 30.0% | 35.8% |
| 19.00 pm - 21.00 | 67.1% | 65.3% | 62.1% | 23.5% | 70.0% | 62.7% |
| After 21.00 | 1.3% | 0.0% | 6.9% | 0.0% | 0.0% | 1.5% |
| **Dinner outside house** | 1.3% | 2.0% | 0.0% | 0.0% | 0.0% | 1.0% |
| **Resting outside after dinner** | 2.6% | 6.1% | 6.9% | 0.0% | 0.0% | 3.5% |
| **Go outside at night** | 27.6% | 20.4% | 34.5% | 64.7% | 73.3% | 36.8% |
| Go to neighborhoods | 68.2% | 81.8% | 90.9% | 63.6% | 89.2% | 80.4% |
| Go hunting/fishing | 13.6% | 0.0% | 9.1% | 36.4% | 5.4% | 10.9% |
| Others | 18.2% | 18.2% | 0.0% | 0.0% | 5.4% | 8.7% |
| **Go outside > 1 hour** | 66.7% | 90.0% | 60.0% | 100.0% | 54.5% | 70.3% |
| **Using personal repellent outside** | 28.6% | 70.0% | 90.0% | 100.0% | 86.4% | 70.3% |
| **Sleeping time** |  |  |  |  |  |  |
| Before 19.00 | 2.6% | 2.0% | 0.0% | 0.0% | 0.0% | 1.5% |
| 19.00 pm - 21.00 | 44.7% | 20.4% | 10.3% | 11.8% | 43.3% | 30.8% |
| After 21.00 | 52.6% | 77.6% | 89.7% | 88.2% | 56.7% | 67.7% |
| **Waking up time** |  |  |  |  |  |  |
| Before 06.00 | 65.8% | 95.9% | 20.7% | 52.9% | 96.7% | 70.1% |
| 06.00 am - 07.00 | 30.3% | 4.1% | 69.0% | 47.1% | 3.3% | 26.9% |
| After 07.00 | 3.9% | 0.0% | 10.3% | 0.0% | 0.0% | 3.0% |
| **Sleep outside house** | 0.0% | 0.0% | 0.0% | 0.0% | 0.0% | 0.0% |
| **Sleep under ITNs** | 88.2% | 83.7% | 100.0% | 94.1% | 83.3% | 88.6% |
| 1. **Malaria Knowledge, Attitude and Practices (KAP)** | | | | | | |
|  |  |  |  |  |  |  |
| **Heard about malaria** | 76 (100.0%) | 45 (91.8%) | 27 (93.1%) | 17 (100.0%) | 29 (96.7%) | 194 (96.5%) |
| Health center | 18.4% | 64.4% | 3.7% | 0.0% | 86.2% | 35.2% |
| Health workers (included cadres) | 93.4% | 91.1% | 96.3% | 100.0% | 48.3% | 86.2% |
| Poster | 1.3% | 0.0% | 0.0% | 0.0% | 17.2% | 3.1% |
| Television | 0.0% | 8.9% | 0.0% | 0.0% | 0.0% | 2.0% |
| Radio | 0.0% | 0.0% | 3.7% | 0.0% | 0.0% | 0.5% |
| Family member | 1.3% | 2.2% | 0.0% | 0.0% | 6.9% | 2.0% |
| Neighborhood | 6.6% | 22.2% | 0.0% | 0.0% | 44.8% | 14.3% |
| Religious community | 0.0% | 0.0% | 0.0% | 0.0% | 3.4% | 0.5% |
| **malaria is a dangerous disease** | 96.9% | 100.0% | - | - | - | 98.0% |
| **Animal that can bring malaria** |  |  |  |  |  |  |
| Rat | 0.0% | 0.0% | 0.0% | 0.0% | 0.0% | 0.0% |
| Cat | 0.0% | 0.0% | 0.0% | 0.0% | 0.0% | 0.0% |
| Dog | 0.0% | 0.0% | 0.0% | 5.9% | 0.0% | 0.5% |
| Mosquito | 100.0% | 98.0% | 100.0% | 94.1% | 100.0% | 99.0% |
| Fly | 0.0% | 0.0% | 0.0% | 0.0% | 0.0% | 0.0% |
| Cockroach | 0.0% | 0.0% | 0.0% | 0.0% | 0.0% | 0.0% |
| Don't know | 0.0% | 2.0% | 0.0% | 0.0% | 0.0% | 0.5% |
| **How malaria can be transmitted to a person** |  |  |  |  |  |  |
| An unsanitary home | 0.0% | 2.0% | 0.0% | 0.0% | 0.0% | 0.5% |
| Work hard | 0.0% | 0.0% | 3.4% | 0.0% | 3.3% | 1.0% |
| Drinking dirty water/eating contaminated food | 0.0% | 0.0% | 13.8% | 0.0% | 0.0% | 2.0% |
| Bite of a mosquito infected with malaria | 100.0% | 98.0% | 75.9% | 100.0% | 93.3% | 95.0% |
| Don't know | 0.0% | 0.0% | 6.9% | 0.0% | 3.3% | 1.5% |
| **Known malaria symptoms** | 3.6 (± 1.6) | 5.5 (± 1.0) | 3.7 (± 1.4) | 2.5 (± 0.9) | 4.9 (± 1.4) | 4.2 (± 1.6) |
| Fever | 76.3% | 75.5% | 79.3% | 47.1% | 96.7% | 77.1% |
| Shaking chills | 90.8% | 100.0% | 69.0% | 82.4% | 96.7% | 90.0% |
| Dizziness | 44.7% | 87.8% | 86.2% | 52.9% | 86.7% | 68.2% |
| Sweating | 18.4% | 57.1% | 27.6% | 0.0% | 23.3% | 28.4% |
| Fatigue | 5.3% | 42.9% | 0.0% | 0.0% | 26.7% | 16.4% |
| Nausea | 73.7% | 93.9% | 37.9% | 35.3% | 83.3% | 71.6% |
| Vomiting | 50.0% | 91.8% | 69.0% | 29.4% | 76.7% | 65.2% |
| Muscle Pains | 2.6% | 0.0% | 0.0% | 0.0% | 3.3% | 1.5% |
| Don't know | 0.0% | 0.0% | 0.0% | 0.0% | 0.0% | 0.0% |
| **Ways for preventing malaria** |  |  |  |  |  |  |
| Burning mosquito coil | 44.7% | 59.2% | 62.1% | 23.5% | 100.0% | 57.2% |
| Burning cow dung/leaves | 9.2% | 4.1% | 0.0% | 0.0% | 30.0% | 9.0% |
| Sleeping under mosquito nets | 64.5% | 81.6% | 44.8% | 70.6% | 63.3% | 66.2% |
| Wear long-sleeve shirt and trousers | 30.3% | 20.4% | 10.3% | 0.0% | 40.0% | 23.9% |
| Draining stagnant water | 25.0% | 22.4% | 0.0% | 0.0% | 23.3% | 18.4% |
| Use mosquito repellent spray | 0.0% | 4.1% | 0.0% | 0.0% | 0.0% | 1.0% |
| Use traditional herbal repellent | 1.3% | 0.0% | 6.9% | 0.0% | 0.0% | 1.5% |
| Cleaning the house yard | 0.0% | 0.0% | 6.9% | 0.0% | 0.0% | 1.0% |
| Using electric fan | 0.0% | 6.1% | 0.0% | 0.0% | 0.0% | 1.5% |
| Do nothing | 0.0% | 0.0% | 0.0% | 5.9% | 0.0% | 0.5% |
| **Why taking these ways of protection from malaria** |  |  |  |  |  |  |
| Free of charge | 76.3% | 63.3% | 100.0% | 100.0% | 83.3% | 79.6% |
| Cheap | 73.7% | 36.7% | 0.0% | 23.5% | 13.3% | 40.8% |
| Smells nice | 5.3% | 2.0% | 0.0% | 0.0% | 0.0% | 2.5% |
| Like the way it looks | 0.0% | 0.0% | 0.0% | 0.0% | 0.0% | 0.0% |
| Better for stopping mosquito bites | 30.3% | 69.4% | 0.0% | 0.0% | 6.7% | 29.4% |
| Easy to use | 30.3% | 69.4% | 0.0% | 0.0% | 50.0% | 35.8% |
| **What time do mosquitoes with malaria bite?** |  |  |  |  |  |  |
| Early evening | 26.3% | 49.0% | 96.6% | 100.0% | 0.0% | 44.3% |
| In the morning | 21.1% | 26.5% | 13.8% | 11.8% | 0.0% | 17.4% |
| During the day | 1.3% | 0.0% | 13.8% | 0.0% | 0.0% | 2.5% |
| At night | 82.9% | 85.7% | 79.3% | 52.9% | 86.7% | 81.1% |
| Don't know | 1.3% | 0.0% | 0.0% | 0.0% | 13.3% | 2.5% |
| **Mosquitoes were bothering at the household** | 98.7% | 98.0% | 96.6% | 100.0% | 96.7% | 98.0% |
| **Mosquitoes were bothering outside the household in the evenings** | 64.5% | 95.9% | 100.0% | 100.0% | 93.3% | 84.6% |
| **Alternative ways to stop mosquito bites** |  |  |  |  |  |  |
| Sleeping under mosquito nets | 90.8% | 95.9% | 69.0% | 100.0% | 93.3% | 90.0% |
| Eating garlic | 0.0% | 2.0% | 0.0% | 0.0% | 0.0% | 0.5% |
| Spraying insecticides on the house walls | 21.1% | 14.3% | 0.0% | 0.0% | 46.7% | 18.4% |
| Making fire and smoke | 13.2% | 26.5% | 17.2% | 0.0% | 33.3% | 18.9% |
| Drinking lots of water | 0.0% | 0.0% | 0.0% | 0.0% | 0.0% | 0.0% |
| Using personal repellent at night | 19.7% | 18.4% | 3.4% | 0.0% | 0.0% | 12.4% |
| Burning mosquito coils | 1.3% | 4.1% | 10.3% | 0.0% | 0.0% | 3.0% |
| Trimming bushes around the house | 22.4% | 26.5% | 3.4% | 0.0% | 43.3% | 21.9% |
| Removing standing water or rainwater from the area around the home | 22.4% | 30.6% | 3.4% | 0.0% | 50.0% | 23.9% |
| Using electric fan | 0.0% | 4.1% | 0.0% | 0.0% | 0.0% | 1.0% |
| Don't know | 0.0% | 0.0% | 3.4% | 0.0% | 3.3% | 1.0% |
| **Burn mosquito coil last night** |  |  |  |  |  |  |
| Yes | 39.5% | 38.8% | 13.8% | 23.5% | 66.7% | 38.3% |
| No | 60.5% | 61.2% | 82.8% | 76.5% | 33.3% | 61.2% |
| Don't know | 0.0% | 0.0% | 3.4% | 0.0% | 0.0% | 0.5% |
| **Burn animal manure or leaves last night** |  |  |  |  |  |  |
| Yes | 14.5% | 0.0% | 13.8% | 0.0% | 6.7% | 8.5% |
| No | 85.5% | 100.0% | 82.8% | 100.0% | 93.3% | 91.0% |
| Don't know | 0.0% | 0.0% | 3.4% | 0.0% | 0.0% | 0.5% |
| **Know about the IRS** | 29 (38.2%) | 15 (30.6%) | 26 (89.7%) | 0 (0.0%) | 12 (40.0%) | 82 (68.3%) |
| **How does spraying insecticide on the wall protects malaria from people who stay home?** |  |  |  |  |  |  |
| Prevents mosquitoes from resting | 31.6% | 26.5% | 62.1% | - | 33.3% | 32.3% |
| Kills mosquitoes that land on them | 27.6% | 28.6% | 62.1% | - | 26.7% | 30.3% |
| Cleans the walls | 3.9% | 0.0% | 0.0% | - | 23.3% | 5.0% |
| Don’t know | 0.0% | 0.0% | 0.0% | - | 0.0% | 0.0% |
| The answers 1, 2 and 3 are not right | 0.0% | 0.0% | 0.0% | - | 3.3% | 0.5% |
| **Do you pay for the insecticide when sprayed in your house?** |  |  |  |  |  |  |
| Yes | 3.4% | 0.0% | 0.0% | - | 0.0% | 1.2% |
| No | 96.6% | 100.0% | 100.0% | - | 91.7% | 97.6% |
| Don't know | 0.0% | 0.0% | 0.0% | - | 8.3% | 1.2% |
| **How the treated mosquito net prevents malaria** |  |  |  |  |  |  |
| Prevent mosquitoes from biting | 90.8% | 85.7% | 51.7% | 35.3% | 83.3% | 78.1% |
| Kills mosquitoes that land on them | 32.9% | 91.8% | 69.0% | 58.8% | 70.0% | 60.2% |
| Keeps warm inside | 0.0% | 0.0% | 0.0% | 0.0% | 50.0% | 7.5% |
| Don’t know | 3.9% | 4.1% | 13.8% | 11.8% | 13.3% | 7.5% |
| The answers 1, 2 and 3 are not right | 0.0% | 2.0% | 0.0% | 0.0% | 0.0% | 0.5% |
